# Iron Sulfide Nanoparticles Embedded Into a Nitrogen and Sulfur Co-doped Carbon Sphere as a Highly Active Oxygen Reduction Electrocatalyst

**DOI:** 10.3389/fchem.2019.00855

**Published:** 2019-12-12

**Authors:** Haitao Wang, Xiaoyu Qiu, Wei Wang, Lipei Jiang, Hongfang Liu

**Affiliations:** ^1^Key Laboratory for Green Chemical Process (Ministry of Education), School of Chemistry and Environmental Engineering, Wuhan Institute of Technology, Wuhan, China; ^2^Key Laboratory of Material Chemistry for Energy Conversion and Storage (Ministry of Education), School of Chemistry and Chemical Engineering, Huazhong University of Science and Technology, Wuhan, China; ^3^School of Chemistry and Chemical Engineering, Hunan Institute of Science and Technology, Yueyang, China

**Keywords:** facile synthesis strategy, iron sulfide nanoparticles, carbon spheres, nitrogen and sulfur dual-doping, oxygen reduction

## Abstract

The unique micro/mesoporous spherical nanostructure composed of non-noble metal nanoparticles encapsulated within a heteroatom-doped carbon matrix provides great advantages for constructing advanced non-precious oxygen reduction (ORR) electrocatalysts. Herein, a promising oxygen electrocatalyst comprising iron sulfide (Fe_1−x_S) nanoparticles embedded into a nitrogen and sulfur co-doped carbon sphere (Fe_1−x_S/NS-CS) is successfully explored through a simple and fast polymerization between methylolmelamines (MMA) and ammonium ferric citrate (AFC) as well as a high-temperature vulcanization process. Moreover, the proposed polymerization reaction can be finished completely within a very short time, which is useful for large-scale manufacturing. Impressively, the developed Fe_1−x_S/NS-MCS catalyst demonstrates outstanding ORR catalytic activity in terms of a more positive onset and half-wave potential as well, as much a better methanol tolerance and stability, in comparison with that of Pt/C benchmarked catalyst. The remarkable ORR electrocatalytic properties are strongly associated with the favorable characteristic spherical N, the S co-doped porous graphitic carbon nanoskeleton incorporated with the Fe_1−x_S nanoparticle-encapsulation structure.

## Introduction

The oxygen reduction reaction (ORR) is of great importance to cathode reactions in a class of various renewable electricity techniques, including metal-air batteries and proton exchange membrane fuel cells (Liu et al., 2019; Zhang et al., 2019). However, the thermodynamic barrier and sluggish kinetics of ORRs have always hindered the development of these technologies (Guo et al., 2019; Wang et al., 2019b). Therefore, a low-cost and high-efficiency ORR electrocatalyst is the key to the large-scale commercialization of such sustainable green energy technologies (Wang S. et al., 2018; Wang et al., 2019a; Yuan et al., 2019). Until now, noble metals-based ORR electrocatalysts have generally been considered to be the best choice to expedite the ORR process, but the rocketing costs, scarce resources, and poor durability inhibit their more widespread applications (Greeley et al., 2009; Dai et al., 2015). The development of cost-effective alternatives to precious metals as efficient ORR catalysts, therefore, is of great importance; but this development faces several great challenges. Recently, tremendous efforts have demonstrated that the coordination of iron species with N-doped carbon frameworks (Fe/N-C) possess better ORR catalytic activity than simple N-doped carbon matrices. The promising electrocatalytic activity can be ascribed to the synergistic effect between Fe species and the surface nitrogen and carbon (Chen Z. et al., 2011; Jaouen et al., 2011; Kim et al., 2013). In this respect, various Fe compounds, such as oxides, carbides, and nitrides, have been explored as ORR catalysts, including Fe_3_O_4_/N-doped mesoporous carbon spheres (Wang et al., 2017b), Fe_3_C/N-doped carbon nanosheets, and Fe_2_N@N-doped mesoporous graphitic carbon (Xiao et al., 2016; Wang H. et al., 2018). Although, some progress has been made in the study of Fe/N-C catalysts in the past decade, the ORR catalytic performance is still inferior to noble metals-based electrocatalysts.

Fortunately, recent studies have shown that the ORR electrocatalytic properties of Fe/N-C materials can be improved by introducing sulfur into Fe/N-C (Wang et al., 2015), since the electron spin effect results in the change of charge distribution for the carbon framework (Jeon et al., 2013; Wu et al., 2016), thereby improving the electrical conductivity. In addition, the introduction of S species can also combine with transition metals to form a new type of iron sulfide active site, thus further enhancing the ORR catalytic performance (Xiao et al., 2017a,b). For instance, Jin et al. (2018) synthesized the Fe/N/S-CNTs via pyrolysis of hydrazine hydrate and ferrous sulfate-treated ZIF-8. Xiao et al. (2017a) fabricated the Fe_1−x_S/N, S-MGCS catalyst via a two-step pyrolysis and acid-leaching process. However, the above synthetic method is not only tedious and time-consuming, but also involves the use of expensive and dangerous chemicals. Therefore, exploring an environmentally friendly and facile approach to fabricate the S-coordinated Fe/N-C composites is consequently significant, but it remains challenging.

Being mindful of the above ideas, this paper proposes a facile and fast strategy to fabricate iron sulfide (Fe_1−x_S) nanoparticles embedded into a nitrogen and sulfur co-doped carbon sphere (Fe_1−x_S/NS-CS) through a simple and quick polymerization between methylolmelamines (MMA) and ammonium ferric citrate (AFC), as well as the subsequently high-temperature vulcanization process. Importantly, the proposed polymerization reaction can be finished completely within a very short time (7 min), which makes it useful for large-scale manufacturing. Moreover, together with the advantages of the characteristically spherical N, S co-doped a porous graphitic carbon nanoskeleton incorporated with the Fe_1−x_S nanoparticle-encapsulation structure; the resulting Fe_1−x_S/NS-CS demonstrated an outstanding ORR catalytic activity in terms of a more positive onset and half-wave potential, as well as much better methanol tolerance and stability, in comparison with that of the Pt/C benchmarked catalyst.

## Experimental Section

### Synthesis of Fe_1-x_S/NS-CS

Typically, 2.81 g of melamine is added into 5.6 mL of formaldehyde with constant stirring until it forms a homogeneous transparent solution at 65°C. Meanwhile, 0.04 g of AFC and 0.56 g of poly (vinyl alcohol) are dissolved completely in 80 mL of deionized water to form a uniform orange liquid. Then the two solutions are stirred at 60°C to mix homogeneously. Subsequently, 1.3 mL of acetic acid is injected into the above mixed liquid with continuous stirring at 60°C for 7 min to produce the Fe containing nitrogen-rich carbon polymer spheres (Fe-NCPS). Next, 0.35 g of Fe-NCPS sample is annealed at 600°C under N_2_ protection with a ramp rate of 1°C min^−1^ for 1 h to form Fe-containing N-doped carbon spheres (Fe/N-CS). Finally, 0.06 g of the Fe/N-CS sample and 10 g of thiourea are placed in the center and front end of tube furnace, respectively, and they are then heated to 850°C for 1 h with a heating rate of 10°C min^−1^ and an argon flow of 100 sccm. After that, the Fe_1−x_S/NS-CS catalyst is obtained.

### Synthesis of NS-CS, Fe_3_O_4_/NS-CS and N-CS

For comparison, the NS-CS control sample is prepared in a similar way without the addition of AFC. Moreover, the Fe_3_O_4_/N-CS control material is also synthesized by a similar way, just replacing thiourea with urea in the process of synthesis. Additionally, the N-CS control catalyst is obtained through a similar way without adding AFC and replacing thiourea with urea.

## Results and Discussion

**Figure 1:**
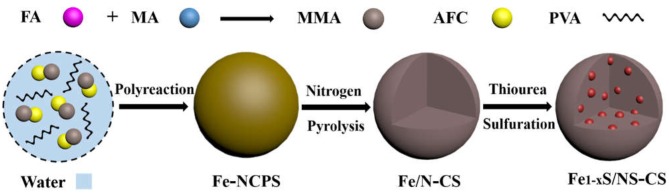
Schematic demonstration of the formation process of Fe_1−x_S/NS-CS.

The Fe_1−x_S/NS-CS catalyst was fabricated via a facile three-step method, including polyreaction, pyrolysis, and a high-temperature vulcanization step, as schematically displayed in Figure 1 (Experimental detail, Supplementary Material). Firstly, a simple hydroxymethylation happened between the formaldehyde (FA) and melamine (MA) molecules, which resulted in the formation of MMA (Ma et al., 2012). Then, the formed MMA species were polymerized with AFC (Wang et al., 2017b) under the catalysis of acetic acid and with the existence of poly (vinyl alcohol) (PVA), ultimately resulting in the Fe-containing nitrogen-rich carbon polymer spheres (Fe-NCPS). It is worth mentioning that the polymerization reaction was finished completely within a very short time (7 min). Thus, the method outlined here is simple and quick to operate, and this makes it useful for large-scale manufacturing. Next, the Fe-NCPS samples were annealed at 600°C under N_2_ protection to form Fe-containing N-doped carbon spheres (Fe/N-CS). Finally, the obtained Fe/N-CS samples were vulcanized into iron sulfide/nitrogen and a sulfur co-doped carbon sphere (Fe_1−x_S/NS-CS) by the pyrolysis of thiourea.

**Figure 2:**
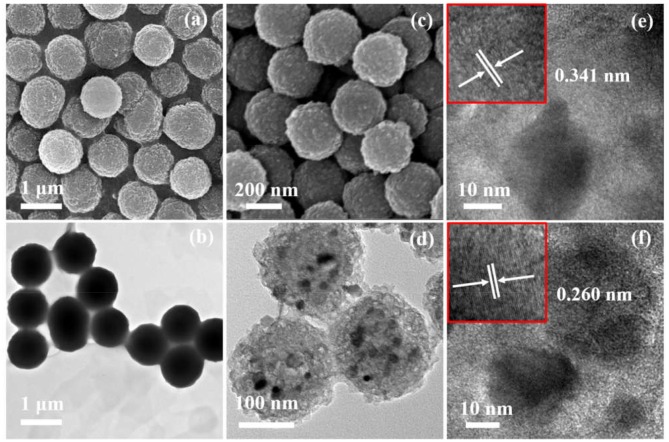
**(a)** SEM image and **(b)** the corresponding TEM image of Fe-NCPS. **(c)** SEM image and **(d)** the corresponding TEM image of Fe_1−x_S/NS-CS. **(e,f)** HRTEM images of Fe_1−x_S/NS-CS.

**Figure 3:**
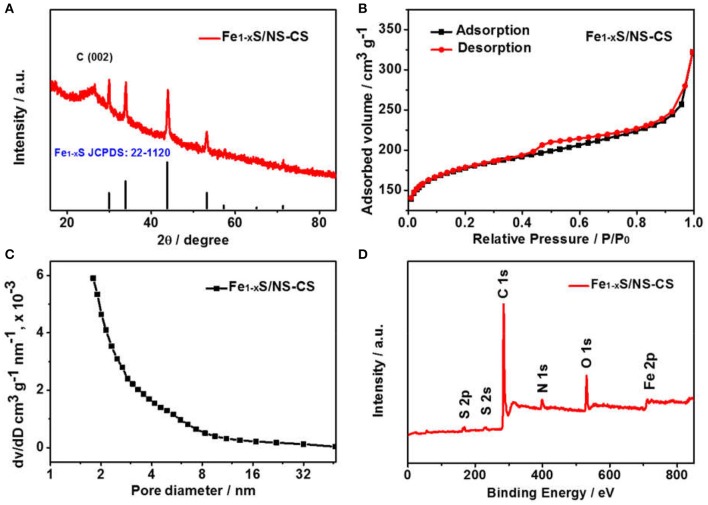
**(A)** XRD patterns of Fe_1−x_S/NS-CS. **(B)** N_2_ sorption isotherms and **(C)** the corresponding pore size distribution (inset) of Fe_1−x_S/NS-CS. **(D)** XPS survey spectra of Fe_1−x_S/NS-CS.

Figure 2 displays the morphologies and microstructures of Fe-NCPS and Fe_1−x_S/NS-CS. It can be seen from Figures 2a,b that the Fe-NCPS have well-defined, solid, spherical morphologies with diameters of ~800 nm. After the pyrolysis process and high-temperature vulcanization step, the similar spherical morphologies are also retained (Figures 2c, 3D). However, the diameter of the carbon spheres obviously decrease from 800 to ~200 nm. Moreover, fair amounts of nanoparticles, indicated by black dots, are seen to be embedded in the spherical carbon skeleton. The XRD result in Figure 3A indicates that the nanoparticles are assigned to the crystalline Fe_1−x_S (JCPDS: 22-1120). In Figure 3A, the peaks located at about 29.9, 33.9, 43.8, and 53.2° are attributed to the crystal planes of (200), (204), (208), and (220) of crystalline Fe_1−x_S (JCPDS: 22-1120), respectively. The result suggests that the iron species in Fe-NCPS samples are transformed into Fe_1−x_S after the pyrolysis and high-temperature vulcanization step. The accurate microstructures of Fe_1−x_S/NS-CS are further investigated by HRTEM. Compared to the solid carbon spheres of Fe-NCPS, the Fe_1−x_S/NS-CS sample not only shows the porous structure, but also displays a large number of Fe_1−x_S nanoparticles (~18 nm) embedded in the sphere (Figure 2d). A detailed examination of Figure 2e shows that the Fe_1−x_S nanoparticles are well-encapsulated by graphitized carbon shells, where the lattice spacing of 0.340 nm corresponds to the (002) plane of graphitic carbon. Additionally, the interplanar lattice spacing of 0.260 nm observed in Figure 2f can be indexed to the (204) plane of crystalline Fe_1−x_S. Peculiarly, such a nanoparticle-encapsulation geometric confinement structure can not only effectively suppress the oxidation, agglomeration, and dissolution of Fe_1−x_S nanoparticles during the ORR catalysis process, but can also activate the interfacial contact between neighboring graphitic carbon layers and Fe_1−x_S nanoparticles, thereby enhancing the ORR electrocatalytic activity and durability (Yang et al., 2010; Candelaria et al., 2012; Wu et al., 2012).

The specific surface area of Fe_1−x_S/NS-CS is assessed by measuring the N_2_ sorption isotherm, and the corresponding pore size distribution is obtained by using the Barrett-Joyner-Halenda (BJH) method. It is noteworthy that the Fe_1−x_S/NS-CS catalyst displays the type II adsorption isotherms with a typical type H4 hysteresis loop (Figure 3B) as this indicates the coexistent of micropores and a mesoporous structure in the Fe_1−x_S/NS-CS. In addition, such pore properties can also be confirmed from the corresponding pore size distribution. It can be seen from Figure 3C that the pore diameters in the Fe_1−x_S/NS-CS are distributed in the range of 1.5 to 18 nm. It is of note that the specific surface area, average pore size, and total pore volume of Fe_1−x_S/NS-CS are calculated to be 628.7 m^2^ g^−1^, 6.67 nm, and 0.50 cm^3^ g^−1^, respectively. The large surface area and abundant micropores and mesopores are expected to expose intensive catalytic active sites and facilitate the efficiency of ORR-related ion diffusion, thus strengthening the ORR electrocatalytic activity (Liang et al., 2014).

**Figure 4:**
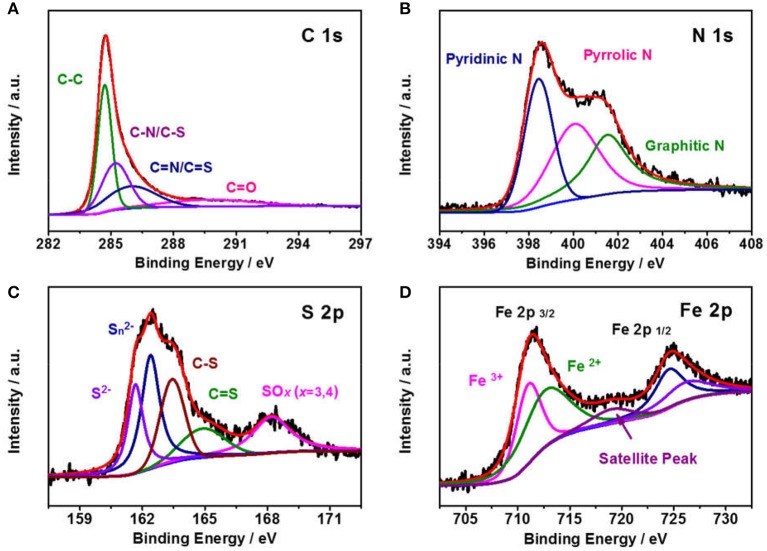
High-resolution XPS spectra of **(A)** C 1s, **(B)** N 1s, **(C)** S 2p, and **(D)** Fe 2p for the Fe_1−x_S/NS-CS.

To probe the elemental compositions and chemical state of Fe_1−x_S/NS-CS, an X-ray photoelectron spectroscopy technic is employed. The full XPS survey spectra in Figure 3D indicates the presence of sulfur, carbon, nitrogen, oxygen, and iron species in the Fe_1−x_S/NS-CS catalyst, and the corresponding surface contents of S, N, O, C, and Fe are 1.71 at %, 5.00 at %, 9.53 at %, 82.73 at %, and 1.04 at %, respectively. The visibility of S 2p and S 2s signals observed in Figure 3D suggest that the sulfur species are successfully introduced into Fe/N-CS after the pyrolysis of thiourea. Figure 4A shows the high-resolution XPS spectrum, where the peaks observed at 285.3 and 286.7 eV are assigned to the C-N/C-S and C = N/C = S (Zhu et al., 2017), demonstrating the N and S atoms are successfully doped into carbon matrices. The high-resolution N 1s spectrum (Figure 4B) reveals the presence of three prominent bands at around 398.4, 400.5, and 401.8 eV, which corresponds to pyridinc N, pyrrolic N, and graphitic N, respectively (Wang et al., 2017a). Figure 4C shows the high-resolution S 2p spectrum of Fe_1−x_S/NS-CS, which can be fitted into five peaks, where the binding energies at 161.8 and 162.6 eV are assigned to the S^2−^ and ${\text{S}}_{\text{n}}^{\;2-}$ of Fe_1−x_S (Bronold et al., 1997; Bukhtiyarova et al., 2000; Xiao et al., 2017a), while the peaks observed at 163.8 and 165.3 eV belonged to the C-S and C = S (Yang et al., 2011). In addition, the peak at the binding energy of 168.6 eV in Figure 4C can be assigned to the oxidized sulfur species (SO_X_) due to the air contact (Wu et al., 2015). The high-resolution Fe 2p spectrum of Fe_1−x_S/NS-CS is displayed in Figure 4D, where the peaks centered at around 712 and 724 eV can be assigned to the Fe 2p_2/3_ and Fe 2p_2/1_ in crystalline Fe_1−x_S (Chen W. et al., 2011). Moreover, the appearance of the satellite peak located at 718.9 eV indicated the co-existence of Fe^2+^ and Fe^3+^ in Fe_1−x_S/NS-CS (Peng et al., 2013), further confirming the formation of Fe_1−x_S.

**Figure 5:**
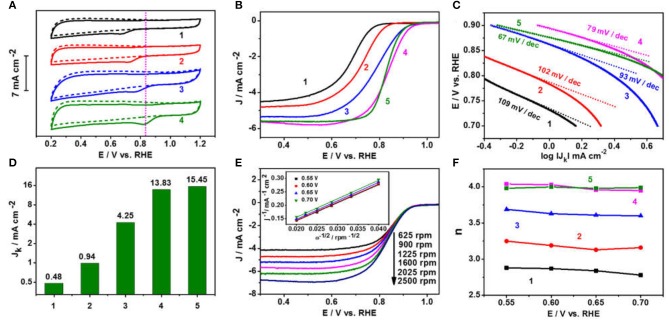
**(A)** CVs in N_2_ or O_2_-saturated 0.1 M KOH. **(B)** LSVs in O_2_-saturated 0.1 M KOH. **(C)** Tafel plots and **(D)** the kinetic current density *J*_*K*_ at the potential of 0.8 V. **(E)** LSVs of Fe_1−x_S/NS-CS at different rotation rates (inset shows the corresponding K-L plots at 0.55–0.70 V). **(F)** The electron transfer number. (1) N-CS, (2) NS-CS, (3) Fe_3_O_4_/N-CS, (4) Fe_1−x_S/NS-CS, and (5) 20% Pt/C.

Motivated by the characteristic spherical nitrogen and sulfur co-doped graphitic carbon nanoskeleton incorporated with the Fe_1−x_S nanoparticle-encapsulation structure, the ORR catalytic activities of Fe_1−x_S/NS-CS were assessed in O_2_-saturated 0.1 M KOH. Before that, the vulcanizing temperature was optimized (Figure S1). For comparison, N-CS, NS-CS, Fe_3_O_4_/N-CS (Figure S2), and commercial 20% Pt/C catalysts were also investigated. Figure 5A shows the cyclic voltammograms (CVs) of all materials in N_2_ or O_2_-saturated 0.1 M KOH, in which the cathodic peak potential of Fe_1−x_S/NS-CS (0.828 V, vs. RHE) is more positive compared to N-CS (0.660 V), NS-CS (0.706 V), and Fe_3_O_4_/N-CS (0.759 V), suggesting pronounced ORR catalytic activity of Fe_1−x_S/NS-CS material. The corresponding linear sweep voltammetrys (LSVs) curves were recorded to further evaluate the excellent ORR catalytic performance of Fe_1−x_S/NS-CS (Figure 5B). Remarkably, the onset potential (E_0_) of the Fe_1−x_S/NS-CS catalyst was 0.989 V, which was more positive than that of Pt/C catalyst (0.973 V) and much more positive than that of N-CS (0.833 V), NS-CS (0.895 V), and Fe_3_O_4_/NS-CS (0.967 V) (Table S1). Moreover, the Fe_1−x_S/NS-CS held the most half-wave potential (E_1/2_ = 0.840 V), which even exceeded the benchmarked 20% Pt/C (0.831 V) and other Fe-based related electrocatalysts previously reported (Table S2). Figure 5C displays a Tafel slope of 79 mV dec^−1^ for the Fe_1−x_S/NS-CS catalyst, which is very close to that of Pt/C (67 mV dec^−1^), highlighting the similar ORR kinetic processes of Fe_1−x_S/NS-CS as commercial Pt/C. The excellent ORR electrocatalytic activity of Fe_1−x_S/NS-CS is further confirmed by the much higher kinetic current density *J*_*K*_, as shown in Figure 5D. The *J*_*K*_ of Fe_1−x_S/NS-CS (13.83 mA cm^−2^) was much higher than that of N-CS (0.48 mA cm^−2^), NS-CS (0.94 mA cm^−2^), and Fe_3_O_4_/NS-CS (4.25 mA cm^−2^) at the potential of 0.8 V. To deeply elucidate the ORR pathway and kinetics, the LSVs of N-CS, NS-CS, Fe_3_O_4_/N-CS, Fe_1−x_S/NS-CS, and 20% Pt/C at different rotation rates (625–2,500 rpm) were recorded (Figure 5E and Figures S2–S5). Meanwhile, the corresponding K-L plots at different potentials (0.55–0.7 V) were also obtained, as shown in Figure 5E and Figures S3–S6. Unlike, the K-L plots of N-CS, NS-CS, Fe_3_O_4_/N-CS, the Fe_1−x_S/NS-CS and 20% Pt/C exhibit good linearity with a similar slope, which is indicative of the first-order ORR kinetics of Fe_1−x_S/NS-CS and 20% Pt/C. Figure 5F displays the electron transfer number (*n*) of N-CS, NS-CS, Fe_3_O_4_/N-CS, Fe_1−x_S/NS-CS, and 20% Pt/C. The average values of *n* at the potential range from 0.55 to 0.70 V for the N-CS, NS-CS, Fe_3_O_4_/N-CS, Fe_1−x_S/NS-CS, and 20% Pt/C were 2.84, 3.18, 3.63, 3.99, and 3.98, respectively, revealing a dominant four-electron ORR catalytic pathway under the electrocatalyst of Fe_1−x_S/NS-CS.

According to above analysis, the Fe_1−x_S/NS-CS sample displayed an efficient ORR activity and pathway, which can be ascribed to several factors. Firstly, the graphitic carbon matrixes contribute to excellent electrical conductivity and stability, thus leading to good electrochemical performances (Xia et al., 2016). Secondly, the N dopants, especially the graphitic N and pyridinic N dopant, modify the electroneutrality and fermi level of neighbor carbon atoms, thus facilitating the adsorption of O_2_ (Wang and Su, 2014). Thirdly, the introduction of S species into the carbon framework had an effect as well, since the S-dopants are beneficial for ORR electrocatalyst through the electron spin effect (Jeon et al., 2013; Wang et al., 2015; Wu et al., 2016). On the score, the improved catalytic activity that can be seen from the electrochemical activity is enhanced from N-CS to NS-CS (Figure 5B). Fourthly, the formed Fe_1−x_S catalytic active substance and the possible synergetic interaction between Fe_1−x_S nanoparticles and the protective N and S co-doped graphitic carbon layer would also contribute to the enhanced activity. In this case, the Fe_1−x_S/NS-CS would hold the most half-wave potential (E_1/2_ = 0.840 V), which is more positive than that of NS-CS and Fe_3_O_4_/N-CS (Figure 5B). The important role of Fe_1−x_S species in improving the ORR electrocatalytic performance has been clearly established. Finally, the large surface area and abundant porous carbon architectures are expected to expose intensive catalytic active sites and facilitate the mass transport efficiency (Liang et al., 2014). Summarily, the efficient ORR activity and pathway of Fe_1−x_S/NS-CS are mainly attributed to the moderate N and S co-doping, the graphitic carbon nanoskeletons with large surface areas and abundant porous architectures, the formed Fe_1−x_S catalytic active substance, and the possible synergetic interaction between Fe_1−x_S nanoparticles and the protective N and S co-doped graphitic carbon layer.

**Figure 6:**
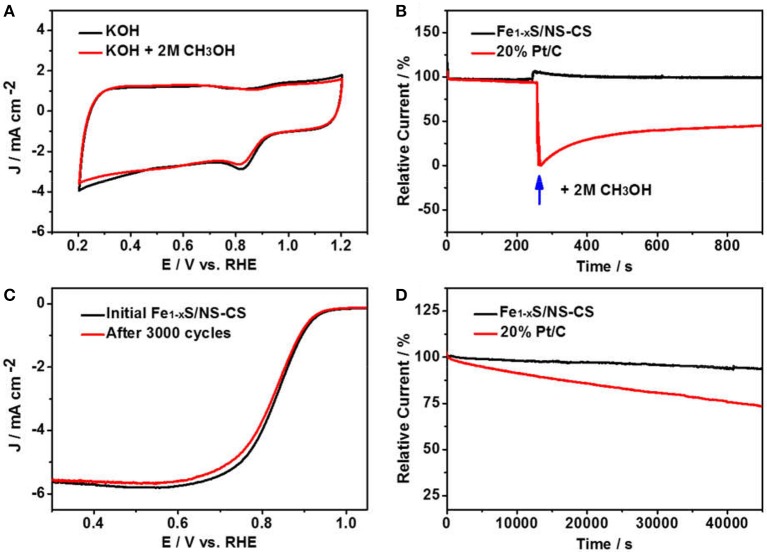
**(A)** CVs of Fe_1−x_S/NS-CS in O_2_-saturated 0.1 M KOH or in the presence of 2 M methanol O_2_-saturated 0.1 M KOH. **(B)** Chronoamperometric responses of Fe_1−x_S/NS-CS and 20% Pt/C in O_2_-saturated 0.1 M KOH with an injection of 2 M methanol. **(C)** LSVs of Fe_1−x_S/NS-CS before and after 3,000 potential cycles. **(D)** Chronoamperometric responses of Fe_1−x_S/NS-CS and 20% Pt/C at 0.6 V over 40,000 s (Relative Current = Measured Current**/**Initial Current).

For application, the outstanding methanol tolerance and stability are also necessary for an ORR electrocatalyst. The methanol resistance effect of Fe_1−x_S/NS-CS was firstly evaluated by cycling the Fe_1−x_S/NS-CS catalyst from 0.2 to 1.2 V in O_2_-saturated 0.1 M KOH with 2 M methanol (Figure 6A), while the 20% Pt/C was also benchmarked (Figure S7). Impressively, the CV curve of Fe_1−x_S/NS-CS has no obvious change in the presence of 2 M methanol. However, for the 20% Pt/C catalysts, a distinct methanol oxidation peak appeared. Additionally, the effect of the methanol crossover for the Fe_1−x_S/NS-CS and 20% Pt/C were also evaluated by the chronoamperometric response. As displayed in Figure 6B, there is no noticeable current attenuation for Fe_1−x_S/NS-CS after injecting CH_3_OH as compared to 20% Pt/C, demonstrating that the developed Fe_1−x_S/NS-CS material possesses excellent tolerance to methanol crossover. Figures 6C,D exhibit the results of several stability performance tests for the Fe_1−x_S/NS-CS and Pt/C. Remarkably, the LSV curve of Fe_1−x_S/NS-CS displays a negligible catalytic activity loss after 3,000 potential cycles (Figure S8). Moreover, the current loss for the Fe_1−x_S/NS-CS catalyst was only about 6.4% after continuous operation for 45,000 s, whereas 20% Pt/C displayed a more rapid current loss. The above results convincingly showed that the Fe_1−x_S/NS-CS not only holds an outstanding ORR catalytic performance but also possesses strong methanol tolerance and excellent ORR catalytic stability.

In summary, an excellent ORR catalyst, in which iron sulfide (Fe_1−x_S) nanoparticles were embedded into a nitrogen and sulfur co-doped carbon sphere (Fe_1−x_S/NS-CS), has been successfully explored through a simple and fast polymerization between MMA and AFC as well as a subsequent high-temperature vulcanization process. Compared to the commercial Pt/C catalyst, the resulting Fe_1−x_S/NS-CS demonstrated a superior ORR catalytic performance and methanol tolerance together with much better stability. Therein, the moderate N and S co-doping, the graphitic carbon nanoskeletons with large surface areas and abundant porous architectures, formed Fe_1−x_S catalytic active substance, and the possible synergetic interaction between Fe_1−x_S nanoparticles and the protective N and S co-doped graphitic carbon layer further improve the outstanding electrocatalytic properties of Fe_1−x_S/NS-CS. In consideration of the simple and fast synthetic preparation, the strategy proposed here can potentially be employed for the synthesis of other non-noble metal-based catalysts in large-scale industrial production.

## Data Availability Statement

All datasets generated for this study are included in the article/Supplementary Material.

## Author Contributions

All authors contributed to the writing of the manuscript and have given approval to the final version of the manuscript.

### Conflict of Interest

The authors declare that the research was conducted in the absence of any commercial or financial relationships that could be construed as a potential conflict of interest.

## Supplementary Material

Supplementary data associated with this article—physical characterization and electrochemical measurements details, XRD patterns, and electrochemical measurement data—are available in the Supplementary Material.
