# Supplementary material for: Iron Sulfide Nanoparticles Embedded Into a Nitrogen and Sulfur Co-doped Carbon Sphere as a Highly Active Oxygen Reduction Electrocatalyst
Source: Front Chem. 2019 Dec 12;7:855. doi: 10.3389/fchem.2019.00855 (PMC6920104; doi:10.3389/fchem.2019.00855)
Supplement: Supplementary file 1 [file Data_Sheet_1.docx]

**Supplementary Material**

**Iron Sulfide Nanoparticles Embedded Into a Nitrogen and Sulfur Co-doped Carbon Sphere as a Highly Active Oxygen Reduction Electrocatalyst**

*Haitao Wang ^1*^, Xiaoyu Qiu ^2^, Wei Wang ^3^, Lipei Jiang ^2^, and Hongfang Liu ^2*^*

*^1^* *Key Laboratory for Green Chemical Process (Ministry of Education), School of Chemistry and Environmental Engineering, Wuhan Institute of Technology (WIT), 693 Xiongchu Avenue, Wuhan 430073, P. R. China*

*^2^* *Key laboratory of Material Chemistry for Energy Conversion and Storage (Ministry of Education), School of Chemistry and Chemical Engineering, Huazhong University of Science and Technology (HUST), 1037 Luoyu Road, Wuhan 430074, P. R. China*

*^3^* *School of Chemistry and Chemical Engineering, Hunan Institute of Science and Technology (HNIST), 439 Xueyuan Road, Yueyang, 414006, P. R. China*

*** Corresponding Author**

Tel: +8613437173246, E-mail: [wanghaitao@wit.edu.cn](mailto:wanghaitao@wit.edu.cn) (H. Wang).

Tel: +8613638673026, E-mail: [liuhf@hust.edu.cn](mailto:liuhf@hust.edu.cn) (H. Liu).

**1. Physical characterization**

The surface morphology of obtained materials were studied by field emission scanning electron microscopy (FESEM) using a Nova NanoSEM 450 at an acceleration voltage of 10 kV. The microstructure of all samples were examined by transmission electron microscopy (HRTEM) using a Tecnai G2 20 microscope operating at an acceleration voltage of 200 kV. The crystalline structure of all materials were recorded on Powder X-ray diffraction (XRD) using a Philips PW-1830 X-ray diffractometer with a sweep rate of 5° min^−1^. Additionally, the Raman analysis of obtained materials were carried out using a LabRAM HR800 confocal Raman microscope. The surface chemical components and electronic structures were analyzed by X-ray photoelectron spectroscopy (XPS) using an AXIS-ULTRA DLD-600W instrument. Meanwhile, the Brunauer–Emmett–Teller (BET) surface area of all products were recorded by N_2_ adsorption-desorption isotherm at 77 K using an ASAP 2020 (Micromeritics USA) surface analyzer. The corresponding pore size distributions were calculated according to the Barrett–Joyner–Halenda (BJH) method from the branch of adsorption isotherm.

**2. Electrochemical measurements**

In this study, all the electrochemical measurements were carried out by using an Autolab (CHI760E potentiostat/galvanostat) with the Pine research instrumentation (AFCBP1 biopotentiostat and AFMSRX rotator) in a three-electrode configuration at 25 °C. Therein, a catalyst-coated glassy carbon electrode (GCE, 5 mm in diameter), a graphite rod and a saturated calomel electrode (SCE) were employed as the working electrode, counter electrode and reference electrode, respectively. In particular, the used SCE electrode was protected with salt bridge, which could effectively avoid the penetration of alkaline medium into the SCE electrode. Moreover, all the potential values used in this work were given relative to the RHE scale.

The working electrode was prepared as follows: 5 mg of catalysts were ultrasonically dispersed in a mixture solution including 0.02 mL of 5 wt% nafion solution and 0.98 mL of isopropyl alcohol, to form a homogeneous catalyst ink. Then, 14 μL of catalyst ink was pipetted onto the surface of GCE, yielding a catalyst amount of 0.357 mg cm^-2^. For comparison, a Pt loading (20% Pt/C, ETEK) of 20 μg cm^-2^ was employed in this work.

During the ORR tests, the O_2_ and N_2_ were used to achieve O_2_-rich and O_2_-free electrolyte environments. Cyclic voltammograms (CVs) experiments were conducted in an N_2_ or O_2_-saturated electrolyte at a sweep rate of 50 mV s^-1^. Linear sweeping voltammograms (LSVs) measurements were carried out in O_2_-saturated electrolyte at a scan rate of 5 mV s^-1^. The Koutecky-Levich (K-L) plots at the potentials of 0.55 V-0.70 V were analyzed and fitted into the linear curves, in which the intercepts and slopes could be employed to calculate the kinetic current density (*J_K_*) and number of electrons transferred (n) on the strength of the Koutecky–Levich equations as below:

*J^-1^ = J_L_^-1^ + J_K_^-1^ = B^-1^****ω^-^****^1/2^ + J_K_^-1^* (1)

*B = 0.2nFC_0_D_0_^2/3^****υ****^-1/6^* (2)

*J_K_ = nFkC_0_* (3)

Where *J_L_* and *J* are the diffusion-limiting and overall current densities (mA cm^-2^), ***ω*** is the rotation speed (rpm), *F* is the Faraday constant (*F* = 96 485 C mol^-1^), *C_0_* is the bulk concentration of O_2_ (*C_0_* = 1.21·10^-6^ mol L^-1^), *D_0_* is the O_2_ diffusion coefficient (*D_0_* = 1.9 ·10^-5^ cm^2^ s^-1^), υ is the kinematic viscosity of the electrolyte (υ = 0.01 cm^2^ s^-1^), and k is the electron-transfer rate constant.

During the methanol poisoning and durability measurements, the chronoamperometric response (i-t) curves were acquired at the potential of 0.6 V with a rotation rate of 1600 rpm. In addition, the tolerance of Fe_1-x_S/N, S-CS to methanol was examined by CVs. The stability of Fe_1-x_S/N, S-CS was also assessed by cycling the catalysts between 0.1 V and 1.2 V at a scan rate of 100 mV/s.

**
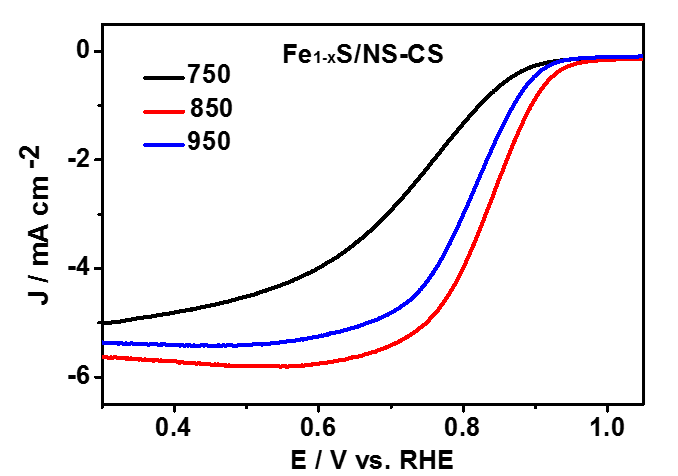
**

**Figure S1.** LSVs of Fe_1-x_S/NS-CS with different vulcanizing temperature.

Noted that the Fe_1-x_S/NS-CS obtained at the vulcanizing temperature of 850 °C exhibits the optimized catalytic activity. Thus, the catalysts discussed in this work are fabricated at the vulcanizing temperature of 850 °C.


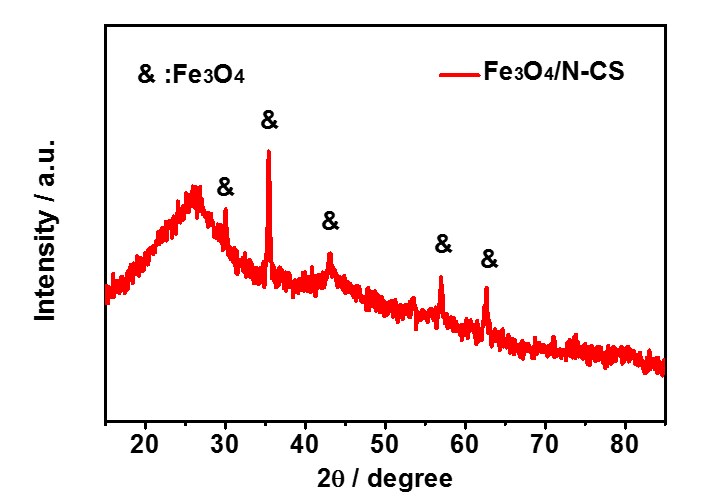


**Figure S2.** XRD pattern of Fe_3_O_4_/N-CS.

**
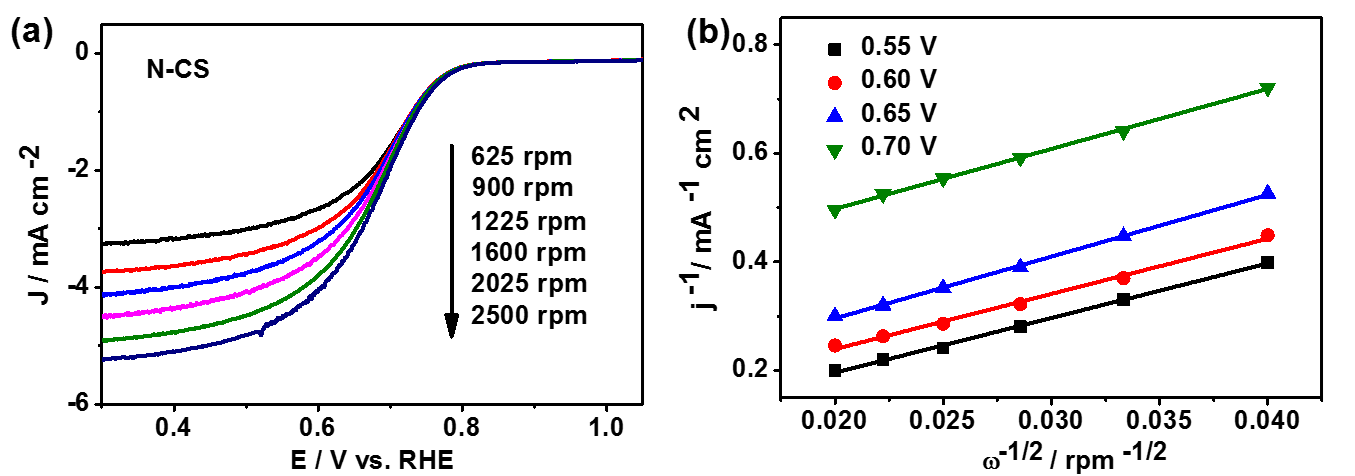
**

**Figure S3.** (a) LSVs of N-CS at different rotation rates and (b) the corresponding K-L plots at the potential range of 0.55 to 0.70V.

**
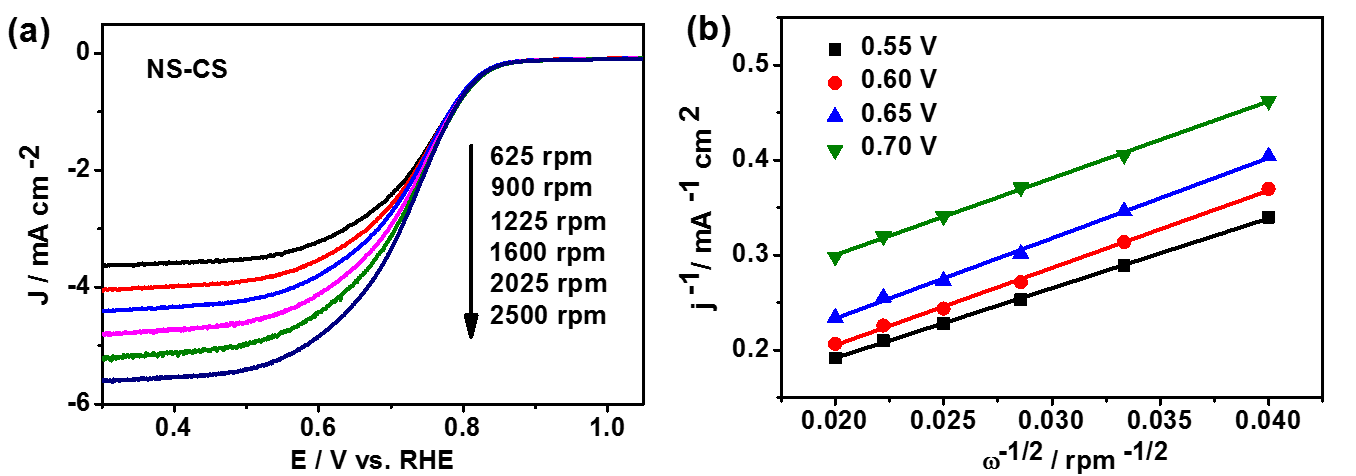
**

**Figure S4.** (a) LSVs of NS-CS at different rotation rates and (b) the corresponding K-L plots at the potential range of 0.55 to 0.70V.

**
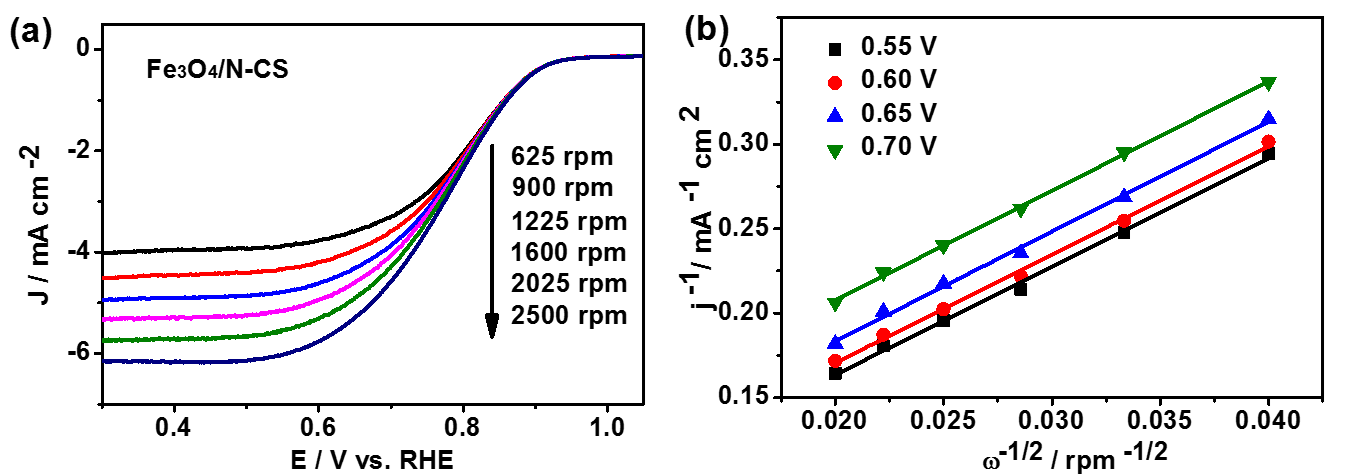
**

**Figure S5.** (a) LSVs of Fe_3_O_4_/N-CS at different rotation rates and (b) the corresponding K-L plots at the potential range of 0.55 to 0.70V.

**
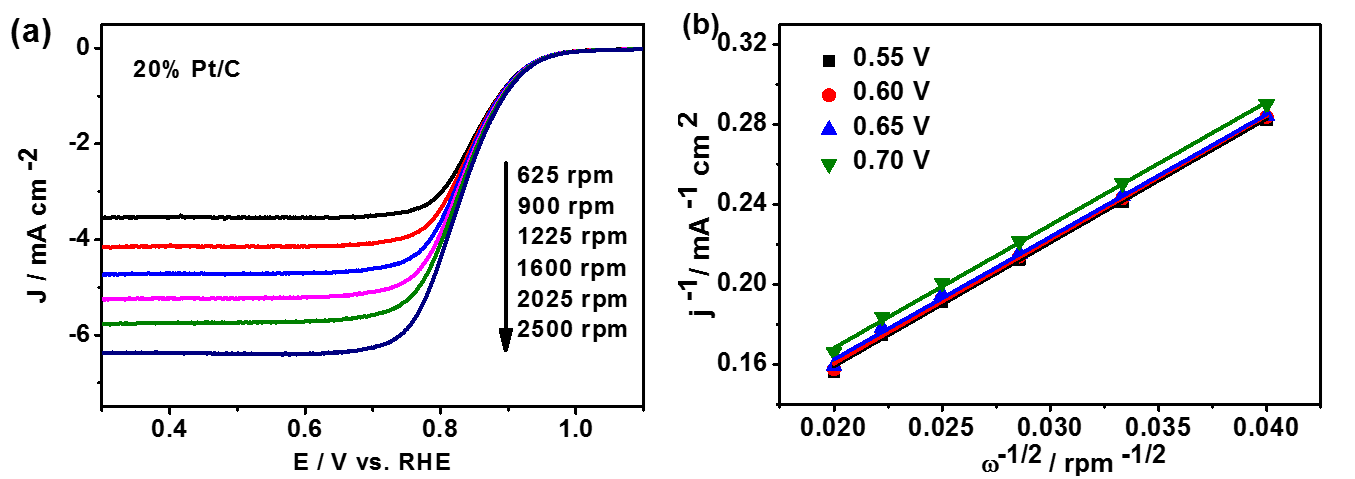
**

**Figure S6.** (a) LSVs of Pt/C at different rotation rates and (b) the corresponding K-L plots at the potential range of 0.55 to 0.70V.

**
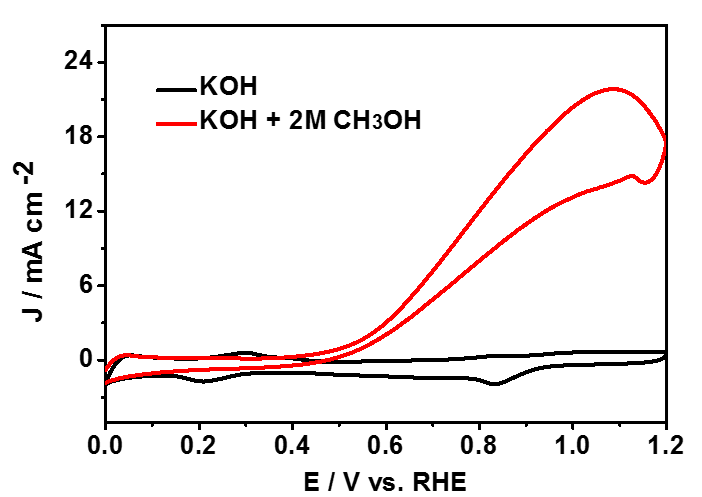
**

**Figure S7.** CVs of 20% Pt/C in O_2_-saturated or in the presence of 2 M methanol O_2_-saturated 0.1 M KOH.

**
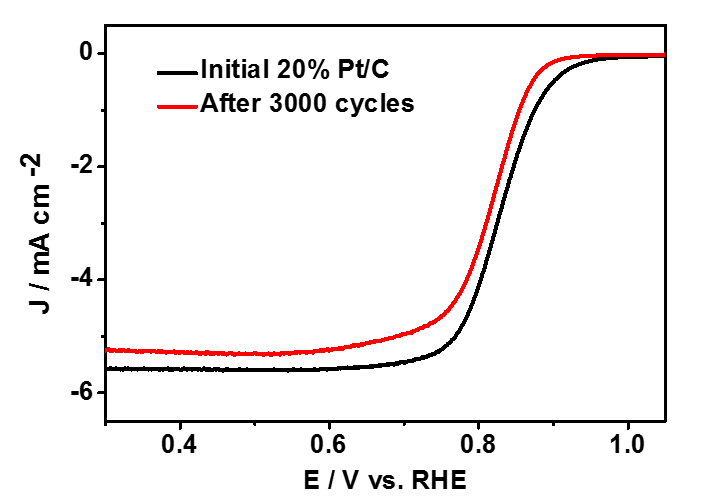
**

**Figure S8.** LSVs of 20% Pt/C before and after 3000 potential cycles.

**Table S1.** Comparison of the ORR catalytic activity of N-CS, NS-CS, Fe_3_O_4_/N-CS and Fe_1-x_S/NS-CS.

| Sample | E_p_ vs RHE (V) | E_0_ vs RHE (V) | E_1/2_ vs RHE (V) | J_i_ (mA cm^-2^) |
| --- | --- | --- | --- | --- |
| N-CS | 0.660 | 0.833 | 0.677 | 4.51 |
| NS-CS | 0.706 | 0.895 | 0.725 | 4.82 |
| Fe_3_O_4_/N-CS | 0.759 | 0.967 | 0.784 | 5.36 |
| Fe_1-x_S/NS-CS | 0.828 | 0.989 | 0.840 | 5.63 |

E_0_: defined as the corresponding potential when ORR current density reaches 0.1 mA cm^-2^.

J_i_: defined as the corresponding current density at the potential of 0.3 V.

**Table S2.** Comparison of the ORR electrocatalytic activity of the Fe_1-x_S/NS-CS with other Fe-based related electrocatalysts previously reported.

| Catalysts | Loading  (mg cm^-2^) | Electrolyte | E_0_ vs RHE (V) | E_1/2_ vs RHE (V) | References |
| --- | --- | --- | --- | --- | --- |
| **Fe_1-x_S/NS-CS** | **0.357** | 0.1 M KOH | **0.989** | **0.840** | **This work** |
| Co_2_FeO_4_/MWCNT | 0.397 | 0.1 M KOH | 0.910 | 0.730 | 1 |
| Fe-SNC-900 | — | 0.1 M KOH | 0.979 | 0.834 | [2](#_ENREF_2) |
| Fe_3_C@C-900 | 0.600 | 0.1 M KOH | 0.980 | 0.800 | 3 |
| C_3_N_4_@NH_2_-MIL-101-700 | 0.429 | 0.1 M KOH | 0.990 | 0.840 | 4 |
| N-doped Fe/Fe_3_C@C | 0.708 | 0.1 M KOH | 0.920 | 0.830 | 5 |
| Fe_3_C@NCNF-900 | — | 0.1 M KOH | 0.980 | 0.840 | 6 |
| Fe-N-CC | 0.100 | 0.1 M KOH | 0.940 | 0.830 | 7 |
| FexP/NPCS | 0.160 | 0.1 KOH | 0.918 | 0.832 | 8 |

**Supplementary References**

1. Wang, J.; Xin, H. L.; Zhu, J.; Liu, S.; Wu, Z.; Wang, D., 3D hollow structured Co_2_FeO_4_/MWCNT as an efficient non-precious metal electrocatalyst for oxygen reduction reaction. *Journal of Materials Chemistry A* **2015**, 3, 1601-1608.

2. Naveen, M. H.; Shim, K.; Hossain, M. S. A.; Kim, J. H.; Shim, Y. B., Template Free Preparation of Heteroatoms Doped Carbon Spheres with Trace Fe for Efficient Oxygen Reduction Reaction and Supercapacitor. *Advanced Energy Materials* **2017,** *7* (5).

3. Kong, A.; Zhang, Y.; Chen, Z.; Chen, A.; Li, C.; Wang, H.; Shan, Y., One-pot synthesized covalent porphyrin polymer-derived core-shell Fe_3_C@carbon for efficient oxygen electroreduction. Carbon 2017, 116, 606-614.

4. Gu, W.; Hu, L.; Li, J.; Wang, E., Hybrid of g-C3N4 Assisted Metal-Organic Frameworks and Their Derived High-Efficiency Oxygen Reduction Electrocatalyst in the Whole pH Range. *Acs Applied Materials & Interfaces* **2016,** *8* (51).

5. Hou, Y.; Huang, T.; Wen, Z.; Mao, S.; Cui, S.; Chen, J., Metal−Organic Framework‐Derived Nitrogen‐Doped Core‐Shell‐Structured Porous Fe/Fe_3_C@C Nanoboxes Supported on Graphene Sheets for Efficient Oxygen Reduction Reactions. *Advanced Energy Materials* **2014,** *4* (11), 1220-1225.

6.  Ren, G.; Lu, X.; Li, Y.; Ying, Z.; Dai, L.; Lei, J., Porous Core–Shell Fe3C Embedded N-doped Carbon Nanofibers as an Effective Electrocatalysts for Oxygen Reduction Reaction. *ACS Applied Materials & Interfaces* **2016,** *8* (6), 4118.

7. Ferrero, G. A.; Preuss, K.; Marinovic, A.; Jorge, A. B.; Mansor, N.; Brett, D. J.; Fuertes, A. B.; Sevilla, M.; Titirici, M.-M., Fe-N-doped carbon capsules with outstanding electrochemical performance and stability for the oxygen reduction reaction in both acid and alkaline conditions. *ACS nano* **2016,** *10* (6), 5922-5932.

8. Hu, K.; Xiao, Z.; Cheng, Y.; Yan, D.; Chen, R.; Huo, J.; Wang, S., Iron phosphide/N, P-doped carbon nanosheets as highly efficient electrocatalysts for oxygen reduction reaction over the whole pH range. *Electrochimica Acta* **2017,** *254*, 280-286.
